# Material stiffness parameters as potential predictors of presence of left ventricle myocardial infarction: 3D echo-based computational modeling study

**DOI:** 10.1186/s12938-016-0151-8

**Published:** 2016-04-05

**Authors:** Longling Fan, Jing Yao, Chun Yang, Zheyang Wu, Di Xu, Dalin Tang

**Affiliations:** Department of Mathematics, Southeast University, Nanjing, 210096 China; Department of Cardiology, First Affiliated Hospital of Nanjing Medical University, Nanjing, 210029 China; Network Technology Research Institute, China United Network Communications Co., Ltd., Beijing, 100048 China; Mathematical Sciences Department, Worcester Polytechnic Institute, 100 Institute Road, Worcester, MA 01609 USA

**Keywords:** Ventricle material property, Prediction, Ventricle model, Ventricle mechanics, Left ventricle

## Abstract

**Background:**

Ventricle material properties are difficult to obtain under in vivo conditions and are not readily available in the current literature. It is also desirable to have an initial determination if a patient had an infarction based on echo data before more expensive examinations are recommended. A noninvasive echo-based modeling approach and a predictive method were introduced to determine left ventricle material parameters and differentiate patients with recent myocardial infarction (MI) from those without.

**Methods:**

Echo data were obtained from 10 patients, 5 with MI (Infarct Group) and 5 without (Non-Infarcted Group). Echo-based patient-specific computational left ventricle (LV) models were constructed to quantify LV material properties. All patients were treated equally in the modeling process without using MI information. Systolic and diastolic material parameter values in the Mooney-Rivlin models were adjusted to match echo volume data. The equivalent Young’s modulus (YM) values were obtained for each material stress–strain curve by linear fitting for easy comparison. Predictive logistic regression analysis was used to identify the best parameters for infract prediction.

**Results:**

The LV end-systole material stiffness (ES-YM_f_) was the best single predictor among the 12 individual parameters with an area under the receiver operating characteristic (ROC) curve of 0.9841. LV wall thickness (WT), material stiffness in fiber direction at end-systole (ES-YM_f_) and material stiffness variation (∆YM_f_) had positive correlations with LV ejection fraction with correlation coefficients r = 0.8125, 0.9495 and 0.9619, respectively. The best combination of parameters WT + ∆YM_f_ was the best over-all predictor with an area under the ROC curve of 0.9951.

**Conclusion:**

Computational modeling and material stiffness parameters may be used as a potential tool to suggest if a patient had infarction based on echo data. Large-scale clinical studies are needed to validate these preliminary findings.

## Background

Determining ventricle tissue material properties and presence of myocardial infarction (MI) noninvasively based on in vivo image data are of great importance in clinical applications. Computational modeling have been widely used in cardiovascular research, adding additional dimensions such as mechanical analysis and predictive methods to precision medicine [1]. Echocardiography is the main imaging modality for left ventricular (LV) structure and function assessment in clinical practice [2–6]. For ventricle material properties and mechanical analysis, Sacks et al. and Humphrey et al. [7, 8] reported biaxial mechanical testing results of passive ventricle tissues. Ventricle fiber architecture and its impact on ventricle mechanical conditions were investigated by Hunter and McCulloch’s group with several significant publications [9–12]. Lee et al. have demonstrated that the fiber orientation estimated by ultrasound elastic tensor imaging was comparable to that measured by magnetic resonance diffusion tensor imaging [13]. Sommer et al. [14] suggested that passive human myocardium can be considered as a nonlinear, anisotropic viscoelastic and history-dependent soft biological material through biaxial extension and triaxial shear testing. Yap et al. [15] have tested rat myocardium with a biaxial tester and 3D ultrasound speckle tracking. Lee et al. [16] made a notable attempt at quantifying fiber orientation in an open chest animal model using shear wave imaging. Couade et al. [17] measured the myocardial stiffness variation with shear wave imaging over the cardiac cycle. Holmes et al. [18] studied functional implications of myocardial scar structure and concluded that large collagen fiber structure is an important determinant of scar mechanical properties. In a more recent paper, Holmes et al. [19] indicated that image-based cardiac mechanical models could provide useful information for clinical and surgical applications. By using magnetic resonance imaging (MRI) and finite element methods, Mojsejenko et al. [20] estimated passive mechanical properties using a porcine infarct model. McGarvey et al. [21] investigated temporal changes in infarct material properties using in vivo MRI and finite element simulations. Xi et al. presented a method for estimating diastolic mechanical parameters of the left ventricle (LV) from cine and tagged MRI measurements and LV cavity pressure recordings, separating the passive myocardial constitutive properties and diastolic residual active tension [22].

There has been huge effort in developing various models to investigate cardiac mechanics with potential clinical applications, including Peskin’s celebrated first ventricle model with moving boundaries using immersed-boundary method [23], the early MRI-based ventricle models for mechanical analysis and investigations by Axel and Saber [24, 25] and the passive and active ventricle modeling by McCulloch et al. including the Continuity package [26–33]. Our group introduced patient-specific cardiac magnetic resonance imaging (CMR)-based right ventricle/left ventricle (RV/LV) models with fluid–structure interactions (FSI) with various surgical designs and potential applications [34–37]. Patient-specific echo-based LV models were introduced to quantify differences in morphology, mechanics and biology between patients with MI and healthy volunteers [38].

In this paper, echo-based 3D LV models and a predictive logistic regression analysis method were introduced to quantify ventricle material properties and identify parameters which may be used to determine the presence of MI. Associations of morphological, material stiffness and mechanical parameters with presence of infarction were investigated.

## Methods

### 3D echo data acquisition

**Fig. 1:**
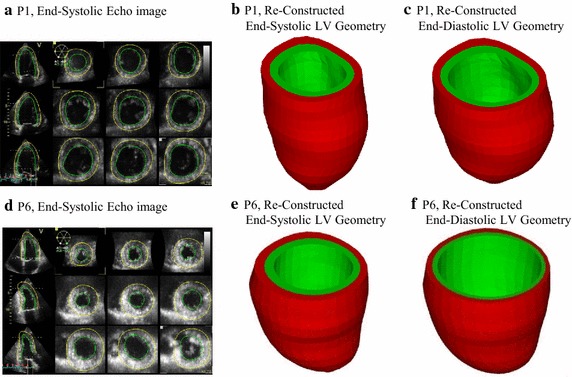
Sample echo images from the Infarct Group (P1) and the Non-Infarcted Group (P6), contours and re-constructed 3D geometries

**Fig. 2:**
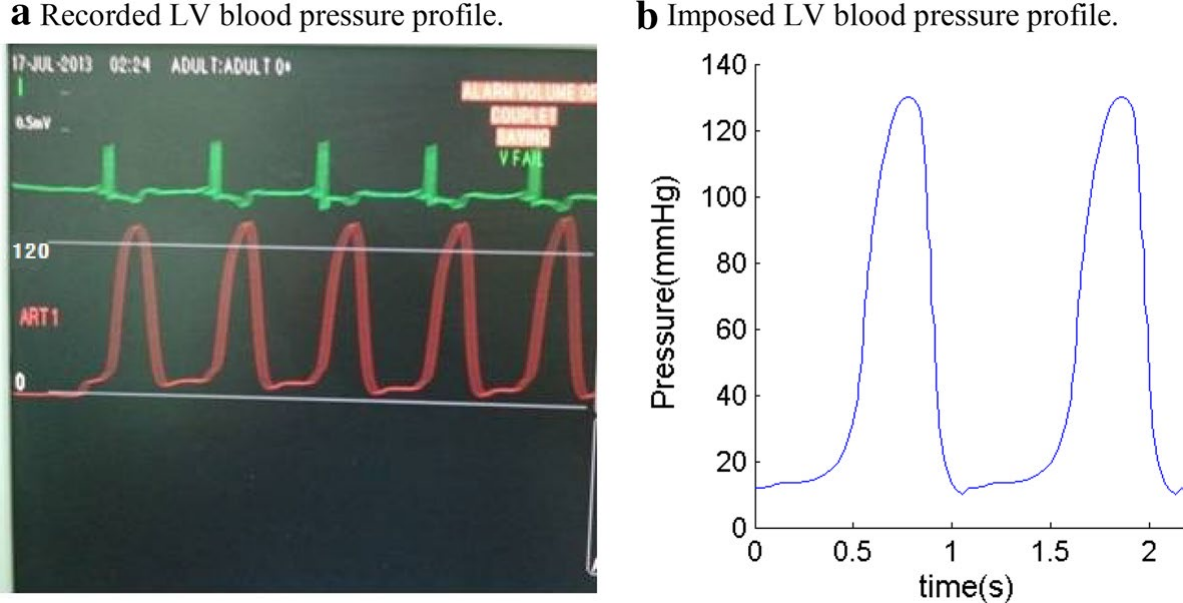
A sample recorded LV pressure profile used in LV model

Patients were recruited to participate in this study at the First Affiliated Hospital of Nanjing Medical University with consent obtained (n = 10, 8 males, mean age 58.3 years). Five patients were with recent infarction (Infarct Group, or IG) and five patients without infarction (Non-Infarcted Group, or NIG). Basic patient information are given in Table 1. Data acquisition procedures were previously reported and are omitted to avoid overlapping [38]. Figure 1 shows the echo images and re-constructed 3D LV geometries from representative patients from the two groups, respectively. A recorded in vivo LV pressure profile is given by Fig. 2.

**Table 1:** Patient demographic and ventricle volume data

	Sex	Age	Pressure (mmHg)		Echo Vol (ml)		Echo EF (%)	Patient MI and catheter information
			Min	Max	Min	Max		
Infarct Group								
P1	M	60	10	121	103	176	41.48	Inferior and posterior MI, 90 % stenosis LAD; 80 % stenosis LCX, 100 % stenosis mid MCA
P2	F	72	8	96	50	98	48.98	Anterior myocardial infarction, 30 % stenosis proximal LAD,100 % in the proximal-mid LAD, LCX, RCA
P3	M	73	9	105	115	193	40.41	Inferior and posterior MI, 90 % stenosis proximal and distal LAD; 90 % stenosis proximal LCX, 99 % stenosis in mid LCX, 50 % stenosis in the mid RCA, total occlusion of distal RCA
P4	M	71	10	120	134	228	41.23	Anterior myocardial infarction, apical left ventricular aneurysm;), 100 % stenosis proximal LAD, 40 % stenosis proximal-mid LCX, 40 % stenosis in the mid RCA
P5	M	58	9	110	70	147	52.38	anterior myocardial infarction, 40 % stenosis in LM, total occlusion of proximal LAD, 80 % stenosis mid LCX, 40 % stenosis mid RCA, total occlusion distal RCA
Mean ± SD		66.8 ± 7.19	9.2 ± 0.84	110.4 ± 10.5	94.4 ± 34.0	168.4 ± 49.1	44.9 ± 5.4	
Non-Infarcted Group								
P6	M	48	9	115	46	116	60.34	
P7	F	43	10	130	46	120	61.67	
P8	M	59	10	118	33	79	58.23	
P9	M	43	9	115	51	120	57.5	
P10	M	56	10	138	46	121	61.98	
Mean ± SD		49.8 ± 7.40	9.6 ± 0.55	123.2 ± 10.3	44.4 ± 6.7	111.2 ± 18.1	59.9 ± 0.02	
P value*		0.06	0.40	0.09	0.01	0.04	0.0004	

P1–P5 are patients with recent infarction. P6–P10 are people without infarction

*F* female, *M* male, *EF* ejection fraction

* P value comparing Infarct Group with Non-Infarcted Group. p < 0.05 indicates the difference was statistically significant

### Two-layer anisotropic LV model construction with fiber orientations

Modeling procedure was previously reported [37, 38] and some essential details are provided here for easy reading. LV material properties were assumed to be hyperelastic, anisotropic, nearly-incompressible and homogeneous. The two groups were treated the same other than that material parameter values were determined for each patient to match echo volume data. Infarct information were not included in patient-specific material models. Standard governing equations and boundary conditions for the LV model were the same as those given in Tang et al. [36, 37] and are given here for completeness

1 $$ \rho {\kern 1pt} v_{{i,{\kern 1pt} \,t\,t}} = \sigma_{i\,j,\,j} \;,\;i,j = 1,2,3;\;{\text{sum}}\;{\text{over}}\;j, $$

2 $$ \varepsilon_{i\,j} = {{(v_{i,\,\,j} + v_{j,\,\,i} + v_{\alpha ,\,\,i} v_{\alpha ,\,\,j} )} \mathord{\left/ {\vphantom {{(v_{i,\,\,j} + v_{j,\,\,i} + v_{\alpha ,\,\,i} v_{\alpha ,\,\,j} )} 2}} \right. \kern-0pt} 2},\;\;i,j,\alpha = 1,2,3, $$

where $\sigma$ is the stress tensor, $\varepsilon$ is the strain tensor, $v$ is displacement, and $\rho$ is material density. The normal stress was assumed to be zero on the outer (epicardial) LV surface and equal to the pressure conditions imposed on the inner (endocardial) LV surfaces.

**Fig. 3:**
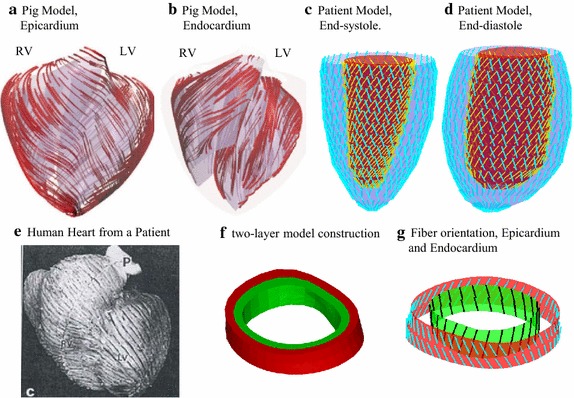
Two-layer model construction with fiber orientations

The nonlinear Mooney–Rivlin (M–R) model was used to describe the nonlinear anisotropic material properties. The strain energy function for the anisotropic modified M–R model is given by Tang et al. [35–37]:

3 $$ {\text{W}}= {\text{c}}_{ 1} ( {\text{I}}_{ 1} - 3 )  +{\text{ c}}_{ 2} ( {\text{I}}_{ 2} - 3 ) + {\text{D}}_{ 1} [ {\text{exp}}({\rm D}_{ 2} ( {\text{I}}_{ 1} - 3 ) )- 1 ] +{{{\text{K}}_{ 1} } / { ( 2 {\text{K}}_{ 2} )\, {\text{exp[K}}_{ 2} ( {\text{I}}_{ 4} - 1 )^{ 2} - 1 ]}} , $$

4 $$ {\text{I}}_{1} = \sum {C_{ii} } ,\;\,{\text{I}}_{2} =  1/2 [{\rm I}_{1}^{2} - C_{ij} C_{ij} ], $$

where I_1_ and I_2_ are the first and second strain invariants given by, C = [C_ij_] = F^T^F is the right Cauchy-Green deformation tensor, F = [F_ij_] = [∂x_i_/∂X_j_], (x_i_) is the current position, (X_i_) is the original position, c_i_ and D_i_ are material parameters chosen to match echo data and available literature [7, 26, 37, 39], I_4_ = C_ij_(**n**_f_)_i_ (**n**_f_)_j_, C_ij_ is the Cauchy-Green deformation tensor, **n**_f_ is the fiber direction, K_1_ and K_2_ are material constants. We also demonstrated that parameter values can be chosen to match the Fung-type models given in McCulloch et al. [32]:

5 $$ {\text{W}} = \frac{\text{C}}{ 2}(e^{Q} - 1), $$

6 $$ Q = b_{1} E_{ff}^{2} + b_{2} (E_{cc}^{2} + E_{rr}^{2} + E_{cr}^{2} + E_{rc}^{2} ) + b_{3} (E_{fc}^{2} + E_{cf}^{2} + E_{fr}^{2} + E_{rf}^{2} ) , $$

where *E*_*ff*_ is fiber strain, *E*_*cc*_ is cross-fiber in-plane strain, *E*_*rr*_ is radial strain, and *E*_*cr*_*E*_*fr*_ and *E*_*fc*_ are the shear components in their respective coordinate planes, C, b_1_, b_2_, and b_3_ are parameters to be chosen to fit experimental data. In this paper, for simplicity, time-dependent parameter values C in Eq. (5) were chosen to fit echo-measured LV volume data while b1, b2, and b3 were kept as constants for all time steps and for all patients. This will simplify our material comparison analysis. Fiber orientation used data in available literature [10, 34] and two-layer construction were handled the same way as in [37, 38]. Finer orientation angles (see Fig. 3) were set at −60° and 80° for epicardium (outer layer) and endocardium (inner layer) according to the pig model in [10], respectively. Figure 3 shows that fiber orientations from the pig and the human sample followed similar angles and patterns.

It should be noted that the modified M–R model is available on Adina so it was used as our material model. However, the M–R model uses the global coordinate system. For different fiber orientation, the material coefficients in the M–R model have different values. So M–R model is not convenient for us to present parameter values for a given ventricle. Fung-type model Eqs. (5) and (6) uses local fiber coordinate system and the parameter values are independent of fiber orientations. So it is more convenient to use Eqs. (5) and (6) to present and compare ventricle tissue material properties. Parameter values in Eqs. (5) and (6) were chosen to fit the M–R model (which was determined by echo data) using Least-squares method and then used for material comparisons.

### Modeling active contraction and expansion by material stiffening and softening

Since active LV contraction and relaxation are very complex and involve change of sarcomere zero-stress length which is hard to model, some model simplifications are needed to obtain proper models to serve our purposes. McCulloch et al. have introduced active tension in their sophisticated multiscale ventricle models with good success [28–30]. Tang et al. introduced LV/RV models with fluid–structure interactions using material stiffness variations to handle active contraction and relaxation [34–37]. Both active tension and stiffness variation approaches involved adding additional terms in tissue material strain energy functions.

It is commonly accepted that a cardiac cycle may be divided into 4 phases: (1) filling (diastole) phase when blood comes in and fills LV; (2) isovolumic contraction; (3) ejection (systole) phase when blood gets pumped out of LV; (4) isovolumic relaxation. For simplicity, we combined (1) and (2) into our “filling phase” and (3) and (4) into our “ejection” phase. So our model includes two phases only: filling and ejection phases. LV volume, pressure, stress and strain increase from minimum to maximum during our filling phase, and decrease from maximum to minimum during our ejection phase. If we call our filling and ejection phases as our model-defined diastole and systole phases, the terms “end-systole” and “end-diastole” will be the same as the terms “begin-filling” and “begin-ejection” since our filling and ejection phases are directly following each other. It is in this sense end-systole and end-diastole are used in this paper.

Active contraction and expansion were modeled by material stiffening during contraction and material softening during expansion. Our material stiffening and softening approach is similar to that of McCulloch et al.’ active tension approach in a sense both approaches adjust strain energy functions to achieve active contraction and relaxation. Material stiffening and softening were achieved by adjusting parameter values in the material models at each echo-time step (28 echo frames per cardiac cycle) to simulate active contraction and expansion and match LV volume data. For simplicity, we set b_1_ = 0.8552, b_2_ = 1.7005, b_3_ = 0.7742 in Eq. (6) and the value for C in Eq. (5) was adjusted to match echo volume data. The least-squares method was used to find the equivalent Young’s moduli (YM) for the material curves for a chosen stretch interval [1.0–1.3].

A pre-shrink process and geometry-fitting technique for mesh generation were used in our model construction as described in [37, 38]. Under in vivo condition, the ventricles were pressurized and the zero-load ventricular geometries were unknown. An iterative pre-shrink process was applied to the in vivo minimum volume ventricular geometry to obtain the zero-load geometry so that when in vivo pressure was applied, the ventricle would regain its in vivo geometry. Shrinking is achieved by shrinking each slice (short-axis direction) by a shrinking rate and by reducing the slice distances (long-axis direction). However, if the slice was shrunk uniformly, the ventricle wall volume (the muscle) would become smaller, which should not happen. So the inner contour (inner wall of the ventricle) was shrunk more, the outer contour (ventricle outer wall) was shrunk a little less (rate was determined by volume conservation). We started with a 2 % shrinkage (varies with the minimum LV pressure for each patient) and material parameter values from our ex vivo direct biaxial mechanical test data and previous simulations [38], construct the model, and apply the minimum pressure to see if the pressurized LV volume matches the in vivo volume. If not, we adjust the shrinkage and material parameter values, re-made the model, pressurize it and check again. The process is repeated until LV volume matches echo volume with error <0.5 %.

Geometry-fitting mesh generation technique was used to generate the mesh for ventricles with irregular geometries. Basically, we cut each “donut” between two slices into 4 volumes (more if the geometry is more irregular. Then ADINA would generate mesh for each small volume. That way, we have the guarantee that the mesh generated would not be too distorted under large deformation. Mesh analysis was performed by decreasing mesh size by 10 % (in each dimension) until solution differences were less than 2 %. The mesh was then chosen for our simulations.

### Solution methods and simulation procedures

**Fig. 4:**
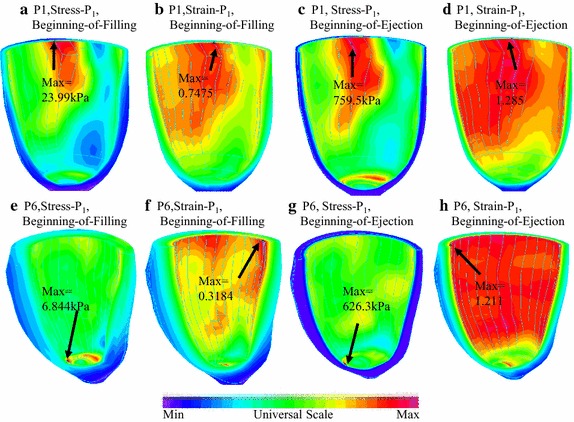
Stress-P_1_ (maximum principal stress) and Strain-P_1_ (maximum principal strain) plots from P1 (Infarct) and P6 (Non-Infarcted) showing stress/strain distribution patterns corresponding to maximum and minimum pressure conditions

The LV models constructed for the 10 patients were solved by a finite element package ADINA (ADINA R&D, Watertown, MA, USA). For each LV data set (11 slices. Slices are short-axis cross sections), we divided each slice into four quarters, each quarter with equal inner wall circumferential length. Ventricle wall thickness, circumferential curvature (C-curvature), longitudinal curvature (L-curvature) and stress/strain were calculated at all nodal points (100 points/slice, 25 points/quarter). The “quarter” values of those parameters were obtained by taking averages of those quantities over the 25 points for each quarter and saved for analysis. The quarter values of those from the two patients were compared to see if there are any statistically significant differences. Formula for curvature calculation can be found in [38]. Maximum principal stress (Stress-P_1_) and strain (Strain-P_1_) were used for analysis and referred to as stress and strain in this paper. Figure 4 shows stress/strain plots from a cut-surface of an LV model, illustrating stress/strain distribution patterns at the beginning-of-ejection and beginning-of-filling phases.

### Statistical analysis

LV wall thickness, volume, diameter (maximum diameter of all slices), height, ejection fraction, C- and L-curvature, stress and strain data, material stiffness parameters and pressure were collected for all patients and standard correlation analyses and student t test were performed for possible correlations and group differences. Logistic regression analysis was used to identify best predictor(s) for ventricle infraction. Sensitivity and specificity of these parameters and their area under the receiver operating characteristic (ROC) curve were determined. A twofold cross-validation procedure was used for model-fitting and prediction. Specifically, we randomly selected 5 out of 10 patients as training data to fit a model that reached the best agreement with actual group category. The remaining patients (test data) were then used to test the model, i.e., the model was used to calculate the probabilities of their group status. The model predictions were compared with the actual group category to obtain the sensitivity and specificity of the predictor. The training and test data were then interchanged and the same procedure was followed to complete a twofold cross-validation. In order to stabilize the result, we repeated the twofold cross-validation 100 times (with random partition of training and testing groups). The probabilities of group assignments from all cross-validation procedures were then combined to calculate the final prediction values. In statistical analysis, all relevant tests were 2-sided. Results were considered statistically significant if P < 0.05. Data analysis was performed using R package [40].

## Results

Correlation and comparison results were presented first. Results for best predictors are given in “Best predictors for patient group category (with or without infarction)” section.

### In vivo LV material stiffness determined by our models

**Fig. 5:**
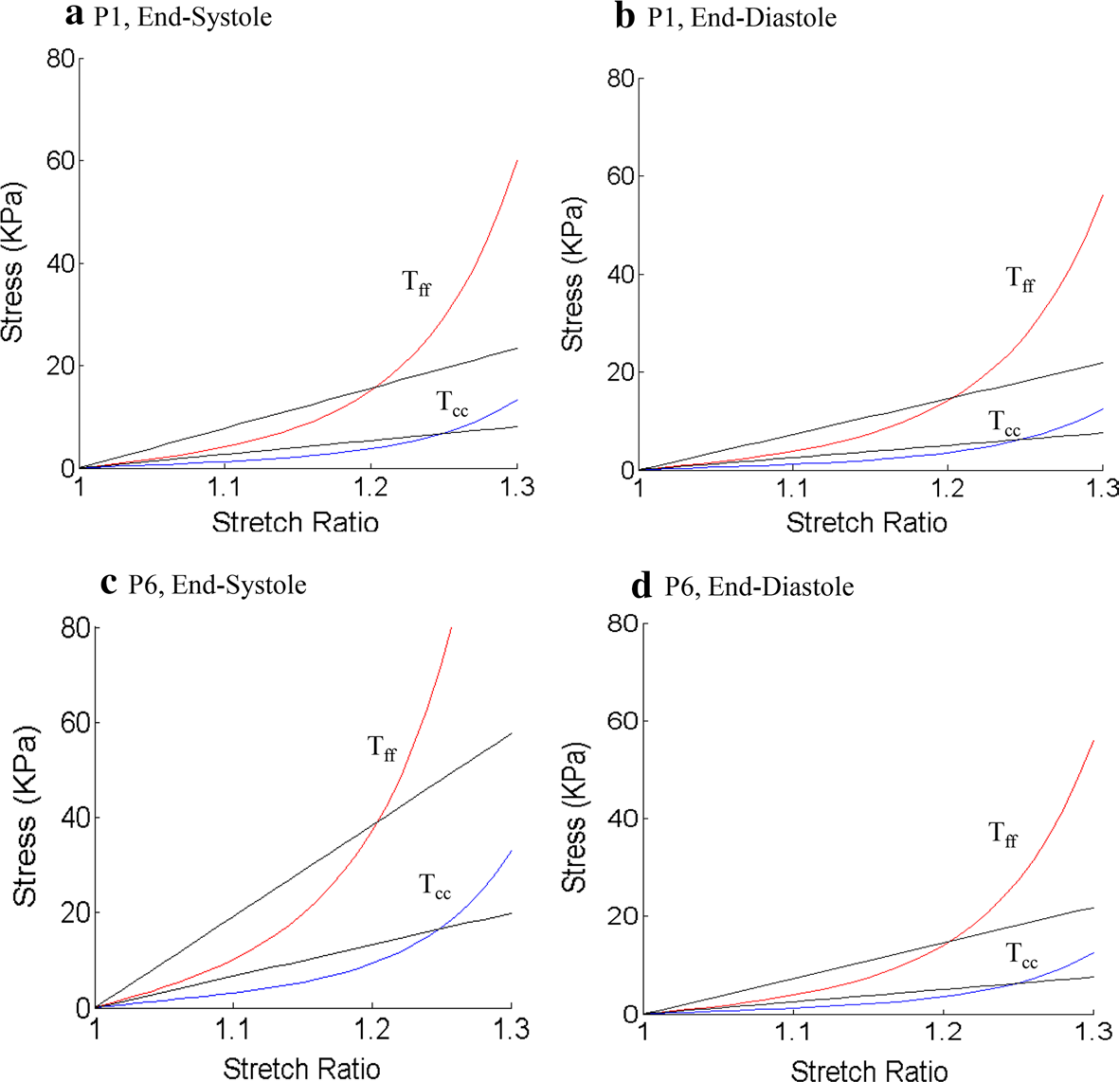
LV material stress–stretch curves from P1 (with infarct) and P6 (without infarct) in fiber coordinates showing P6 tissue stiffness has larger variations between systole and diastole. *T*_*ff*_ stress in fiber direction; *T*_*cc*_ stress in circumferential direction of the fiber

Human ventricle tissue material properties are extremely hard to quantify noninvasively under in vivo conditions. With patient-specific echo ventricle morphological data and the corresponding pressure conditions, we were able to determine parameter values in the M–R model given by Eq. (3) and Fung-type model given by Eqs. (5) and (6). Using the fiber coordinates and Eqs. (5) and (6), end-systole and end-diastole LV material parameter values for the two groups are given in Table 2. Sample stress–stretch plots are given by Fig. 5 for two patients, one from each group for illustration.

**Table 2:** Comparison of material parameters showing the Non-Infarcted Group has greater end-systole YM_f_ and YMi values

	C (kPa)	YM_f_ (kPa)	YM_c_ (kPa)	C (kPa)	YM_f_ (kPa)	YM_c_ (kPa)	YMi (%)
	End of diastole			End of systole			
Infarct Group							
P1	2.5256	72.6	25.1	2.706	77.8	26.9	7
P2	5.2316	150.4	52.0	5.9532	171.2	59.2	14
P3	2.1648	62.2	21.5	2.4354	70.0	24.2	13
P4	1.9844	57.1	19.7	2.255	64.8	22.4	14
P5	2.0746	59.7	20.6	4.3296	124.5	43.0	108
Mean		80.4	27.8		101.66	35.15	31.2
Non-Infarcted Group							
P6	2.5256	72.6	25.1	6.6748	191.9	66.4	164
P7	2.5256	72.6	25.1	7.5768	217.8	75.3	200
P8	3.7884	108.9	37.7	6.8552	197.1	68.2	81
P9	4.059	116.7	40.4	6.8552	197.1	68.2	69
P10	2.5256	72.6	25.1	6.8552	197.1	68.2	171
Mean		88.68	30.68		200.2	69.26	137
P(t test)		0.6939	0.6925		0.00149	0.00147	0.0115

*YM*_*f*_ YM value for the fiber direction, *YM*_*c*_ YM value for circumferential direction of the fiber. ${\text{YMi}} = \frac{{{\text{YM}}_{\text{f}}   {\text{in end of systole}} - {\text{YM}}_{\text{f}}   {\text{in end of diastole}}}}{{{\text{YM}}_{\text{f}}   {\text{in end of diastole}}}}$

### Ventricles with infarct had smaller stiffness variations

Using the mean value of Non-Infarcted Group as the base value, at end-diastole, the mean Young’s modulus (YM) value for the fiber direction (YM_f_) from the two groups were similar (80.4 vs. 88.68 kPa). At end-systole, YM_f_ from the Infarct Group was 49 % smaller than that of the Non-Infarcted Group (101.66 vs. 200.2 kPa).

More interestingly, while the Non-Infarcted Group end-systole YM_f_ (ES-YM_f_) was 126 % higher than its end-diastole value (ED-YM_f_), the Infarct Group ES-YM_f_ was only 26 % higher that its ED-YM_f_. To further explore the impact of LV stiffness variations (∆YM_f_), we define YM index (YMi) as

7 $$ {\text{YMi = }}\frac{{\Delta {\text{YM}}_{\text{f}} }}{{{\text{ED}} - {\text{YM}}_{\text{f}} }}, $$

where ∆YM_f_ = (ES − YM_f_) − (ED − YM_f_). Compared with the Non-Infarcted Group, the Infarct Group had smaller YMi (31.2 vs. 137 %). This indicated that the Non-Infarcted Group ventricles had much better contractility reflected by greater material stiffness variations.

### LV material stiffness variation had best correlation with LV ejection fraction

Table 3 summarizes LV geometrical, material and mechanical stress and strain parameters including WT, Diameter (Dr), Height (Ht), volume, curvature, material stiffness parameters, maximum pressure, stress and strain values for each patient. Correlation analyses were performed to determine whether changes of those parameters were associated with LV ejection fraction (EF). In this cohort, most noticeably, LV material stiffness variation (∆YM_f_ in Table 3) had best correlation with LV EF. Overall, LV EF showed positive correlation with wall thickness (WT), circumferential curvature (C-cur), ES-YM_f_ and ∆YM_f_ with r = 0.8125, 0.7019, 0.9495 and 0.9619, and negative correlation with LV volume and Height (Ht) (r = −0.7882 and −0.6360), respectively. LV EF showed no significant correlation with L-curvature, stress, strain, maximum pressure, ED-YM_f_ and Diameter (Dr).

**Table 3:** Correlations between ejection fraction (EF) and mean values of morphological and mechanical parameters

EF (%)	WT (cm)	C-cur (1/cm)	L-cur (1/cm)	Stress (kPa)	Strain	Vol (ml)	Pmax (mm Hg)	ED-YM_f_	ES-YM_f_	∆YM_f_	Dr (cm)	Ht (cm)
Infarct Group												
41.2	0.44	0.46	0.56	451.0	1.02	175.6	121	72.6	77.8	5.2	7.0	7.4
49.0	0.53	0.60	0.55	227.8	0.88	97.9	96	150.4	171.2	20.8	5.5	7.0
40.2	0.42	0.47	0.24	380.6	1.05	192.6	105	62.2	70.0	7.8	6.8	9.1
41.3	0.55	0.42	0.26	471.8	1.08	227.2	120	57.1	64.8	7.8	7.7	9.0
52.2	0.47	0.48	0.43	383.1	1.07	146.5	110	59.7	124.5	64.8	6.2	8.3
44.8 ± 5.5	0.48 ± 0.05	0.49 ± 0.07	0.41 ± 0.15	382.9 ± 95.6	1.02 ± 0.08	168.0 ± 48.8	110.4 ± 10.5	80.4 ± 39.6	101.7 ± 45.5	21.3 ± 25.1	6.6 ± 0.80	8.1 ± 0.93
Non-Infarcted Group												
60.2	0.76	0.56	0.25	337.3	1.03	115.7	115	72.6	191.9	119.3	6.9	6.6
61.8	0.61	0.60	0.26	413.0	1.05	119.6	130	72.6	217.8	145.2	6.6	6.8
58.2	0.71	0.79	0.35	280.6	1.00	78.8	118	108.9	197.1	88.2	6.1	6.3
57.5	0.75	0.62	0.25	275.3	0.98	119.8	115	116.7	197.1	80.4	6.5	8.1
61.9	0.69	0.59	0.23	432.7	1.08	120.6	138	72.6	197.1	124.5	6.7	7.6
59.9 ± 2.0	0.70 ± 0.06	0.63 ± 0.09	0.27 ± 0.05	347.8 ± 73.0	1.03 ± 0.04	110.9 ± 18.0	123.2 ± 10.3	88.7 ± 22.2	200.2 ± 10.1	111.5 ± 26.8	6.6 ± 0.31	7.08 ± 0.72
R*	0.813	0.702	−0.389	−0.296	0.025	−0.788	0.465	0.196	0.949	0.962	−0.313	−0.636
p*	0.004	0.024	0.266	0.406	0.945	0.007	0.176	0.587	<0.001	<0.001	0.378	0.048

*WT* wall thickness, *C-cur* circumferential curvature, *L-cur* longitudinal curvature, *Vol* left ventricular volume, *ED-YM*_*f*_ end-diastole YM_f_ value, *ES-YM*_*f*_ end-systole YM_f_ value, $\varDelta {\text{YM}}_{\text{f}} = ({\text{ES - YM}}_{\text{f}} ) - ({\text{ED - YM}}_{\text{f}} )$, *Pmax* maximum LV pressure, *Dr* diameter = (max Dx-out + max Dy-out)/2, *Ht* height

* R and p values are for the correlations between EF and the 12 geometric and mechanical parameter values

### Comparison of the two groups in LV WT, curvature and stress/strain using quarter values

The comparison of LV quarterly-averaged wall thickness, circumferential and longitudinal curvature, stress and strain values are given in Table 4. Among the 5 parameters, L-curvature and LV stress showed largest differences. At beginning-of-ejection when LV volume, pressure, stress and strain were at their maxima, the Infarct Group wall thickness and C-curvature were 46 and 31 % lower (thinner), respectively, compared to those of the Non-Infarcted Group. L-curvature and stress from the Infarct Group were 53 and 10 % higher than that from the Non-Infarcted Group. Difference in strain between the two groups was not statistically significant.

**Table 4:** Comparison of quarter mean values of ventricle wall thickness, circumferential curvature, longitudinal curvature and mechanical stress/strain between Infarct and Non-Infarcted Groups

	Qts	WT (cm)	C-Cur (1/cm)	L-Cur (1/cm)	Stress (kPa)	Strain
	Beginning of ejection					
Infarct Group (220 Qts)	Mean	0.4808	0.4860	0.4095	382.85	1.0172
	Stdev	0.1605	0.2559	0.3326	110.68	0.1576
Non-Infarcted Group (220 Qts)	Mean	0.7031	0.6344	0.2684	347.77	1.0273
	Stdev	0.1630	0.4720	0.1933	87.90	0.1285
	P value	8.09E−39	4.89E−05	8.84E−08	0.00026	0.4609
	Beginning of filling					
Infarct Group (220 Qts)	Mean	0.5818	0.6072	0.4595	9.8522	0.4750
	Stdev	0.1436	0.3137	0.3635	4.1702	0.1442
Non-Infarcted Group (220 Qts)	Mean	0.9147	0.9135	0.3195	3.4366	0.2101
	Stdev	0.1393	0.6401	0.3027	0.7047	0.0375
	P value	6.56E−85	4.70E−10	1.42E−05	3.13E−75	1.73E−92

*Qts* quarters, *WT* wall thickness, *C-Cur* circumferential curvature, *L-Cur* longitudinal curvature, *Stdev* standard deviation

At beginning-of-filling when LV volume, pressure, stress and strain are at their minima, the Infartc Group stress, strain and L-curvature were 187,126 and 44 % higher, respectively, than those of the Non-Infarcted Group. Wall thickness and C-curvature from the Infarct Group were 57 and 50 % thinner (lower) than those from the Non-Infarcted Group.

### Best predictors for patient group category (with or without infarction)

Table 5 shows the 6 best combinations (out of 66 possible combinations) of LV parameters that correctly assigned patients to their ultimate outcome group. ES-YM_f_ was the best single predictor among the 12 individual parameters with an area under the ROC curve of 0.9841. The second best single predictor was WT with an area under the ROC curve of 0.9816. The best combination of parameters included WT + ∆YM_f_ an area under the ROC curve of 0.9951.

**Table 5:** Prediction sensitivity, specificity, and ROC values using LV parameters for outcome group prediction by the logistic regression method

Parameter	Probability cut offs	Sensitivity	Specificity	Sensitivity + specificity	Area under ROC	Rank
WT + ∆YM_f_	0.0403	0.9840	0.9800	1.9640	0.9951	1
ES-YM_f_	1.20E−06	0.9200	0.9740	1.8940	0.9841	2
WT	0.9672	0.9060	0.9360	1.8420	0.9816	3
∆YM_f_	1.0000	0.7980	1.0000	1.7980	0.9618	4
WT + ES-YM_f_	2.07E−06	0.9460	0.8520	1.7980	0.9554	5
∆YM_f_ + Stress	0.0013	0.8760	0.8160	1.6920	0.9154	6
C-cur	0.8019	0.7700	0.8400	1.6100	0.7598	
Volume	0.7195	0.6140	0.9760	1.5900	0.7232	
L-cur	0.7898	0.4660	0.8560	1.3220	0.5916	
Height	0.4965	0.5840	0.6200	1.2040	0.5810	
Pmax	0.8249	0.3540	0.8940	1.2480	0.5792	
ED-YM_f_	0.8903	0.2340	0.9840	1.2180	0.5128	
Stress	2.22E−16	1.0000	0.0000	1.0000	0.3742	
Diameter	0.9311	0.0640	0.9460	1.0100	0.3129	
Strain	2.22E−16	1.0000	0.0000	1.0000	0.2641	

## Discussion

### New contribution of this paper

Our previous paper presented our echo-based modeling approach to determine in vivo LV tissue material parameters under using echo data [38]. In this paper, our focus was to identify predictors to differentiate patients with infarct from patients without. In patient screening process, an initial determination about possible infarct based on inexpensive echo method is often needed prior to recommendation of more expensive diagnostic procedures. Unlike the previous paper where infarct region was identified first and then modeled by using different tissue material properties, all patients with and without infarct were treated the same in the modeling process. All 10 LV models assumed that the left ventricle had no infarct. LV material parameters were determined to match echo data for each patient. The information about which patient had infarct was used only in the prediction process for model training and validation.

### Material stiffness parameters as predictors of presence of infarct

The identification of infarct area is of great important in clinical applications. Now that we demonstrated that ventricles with and without infarct have considerably large differences in contractility and material stiffness variations, proper inverse methods could be introduced to determine if a ventricle had infarct based on its contractility and material parameter values predicted by our models. This could serve as the basis for people to develop accurate and automatic methods to identify infarct area based on image data, which is of great clinical relevance.

It should be noted that other imaging modalities (such as magnetic resonance imaging, MRI) may be used to identify infarct. For patients who had done echo test, this method could provide recommendations if the patient should take further steps for diagnosis and proper treatment. MRI is more expensive and insurance policy often require justifications for coverage.

### Model limitations

Our LV models are structure-only models which do not include fluid–structure interactions and do not include ventricle valve mechanics. It was done this way to save modeling labor cost and structure-only models are sufficient for our purpose. Regional material properties were not available because we did not have location-tracking data.

## References

[1] Desmond-Hellmann S, Sawyers CL, Cox DR, Fraser-Liggett C, Galli SJ, Goldstein DB, Hunter D, Kohane IS, Lo B, Misteli T, Morrison SJ, Nichols DG, Olson MV, Royal CD, Yamamoto KR (2011). Toward precision medicine: building a knowledge network for biomedical research and a new taxonomy of disease. National Research Council Committee on a Framework for Development a New Taxonomy of Disease.

[2] Emond M, Mock MB, Davis KB, Fisher LD, Holmes DR, Chaitman BR, Kaiser GC, Alderman E, Killip T (1994). Long-term survival of medically treated patients in the Coronary Artery Surgery Study (CASS) Registry. Circulation.

[3] Møller JE, Hillis GS, Oh JK, Reeder GS, Gersh BJ, Pellikka PA (2006). Wall motion score index and ejection fraction for risk stratification after acute myocardial infarction. Am Heart J.

[4] Quinones MA, Greenberg BH, Kopelen HA, Koilpillai C, Limacher MC, Shindler DM, Shelton BJ, Weiner DH (2000). Echocardiographic predictors of clinical outcome in patients with left ventricular dysfunction enrolled in the SOLVD registry and trials: significance of left ventricular hypertrophy. J Am Coll Cardiol.

[5] Sabia P, Afrookteh A, Touchstone DA, Keller MW, Esquivel L, Kaul S (1991). Value of regional wall motion abnormality in the emergency room diagnosis of acute myocardial infarction: a prospective study using two dimensional echocardiography. Circulation.

[6] Thune JJ, Kober L, Pfeffer MA, Skali H, Anavekar NS, Bourgoun M, Ghali JK, Arnold JM, Velazquez EJ, Solomon SD (2006). Comparison of regional versus global assessment of left ventricular function in patients with left ventricular dysfunction, heart failure, or both after myocardial infarction: the valsartan in acute myocardial infarction echocardiographic study. J Am Soc Echocardiogr.

[7] Sacks MS, Chuong CJ (1993). Biaxial mechanical properties of passive right ventricular free wall myocardium. J Biomech Eng.

[8] Humphrey JD, Strumpf RK, Yin FC (1999). Biaxial mechanical behavior of excised ventricular epicardium. Am J Physiol.

[9] Costa KD, Takayama Y, McCulloch AD, Covell JW (1999). Laminar fiber architecture and three-dimensional systolic mechanics in canine ventricular myocardium. Am J Physiol.

[10] Nash MP, Hunter PJ (2000). Computational mechanics of the heart, from tissue structure to ventricular function. J Elast.

[11] Rogers JM, McCulloch AD (1994). Nonuniform muscle fiber orientation causes spiral Wave drift in a finite element model of cardiac action potential propagation. J Cardiovasc Electrophysiol.

[12] Takayama Y, Costa KD, Covell JW (2002). Contribution of laminar myofiber architecture to load-dependent changes in mechanics of LV myocardium. Am J Physiol Heart Circ Physiol.

[13] Lee WN, Larrat B, Pernot M, Tanter M (2012). Ultrasound elastic tensor imaging: comparison with MR diffusion tensor imaging in the myocardium. Phys Med Biol.

[14] Sommer G, Schriefl AJ, Andrä M, Sacherer M, Viertler C, Wolinski H, Holzapfel GA (2015). Biomechanical properties and microstructure of human ventricular myocardium. Acta Biomater.

[15] Yap CH, Park DW, Dutta D, Simon M, Kim K (2015). Methods for using 3-D ultrasound speckle tracking in biaxial mechanical testing of biological tissue samples. Ultrasound Med Biol.

[16] Lee WN, Pernot M, Couade M, Messas E, Bruneval P, Bel A, Hagège AA, Fink M, Tanter M (2012). Mapping myocardial fiber orientation using echocardiography-based shear wave imaging. IEEE Trans Med Imaging.

[17] Couade M, Pernot M, Messas E, Bel A, Ba M, Hagege A, Fink M, Tanter M (2011). In vivo quantitative mapping of myocardial stiffening and transmural anisotropy during the cardiac cycle. IEEE Trans Med Imaging.

[18] Holmes JW, Nunez JA, Covell JW (1997). Functional implications of myocardial scar structure. Am J Physiol Heart Circ Physiol.

[19] Holmes JW, Costa KD (2006). Imaging cardiac mechanics: what information do we need to extract from cardiac images?. Conf Proc IEEE Eng Med Biol Soc.

[20] Mojsejenko D, McGarvey JR, Dorsey SM, Gorman JH, Burdick JA, Pilla JJ, Gorman RC, Wenk JF (2015). Estimating passive mechanical properties in a myocardial infarction using MRI and finite element simulations. Biomech Model Mechanobiol.

[21] McGarvey JR, Mojsejenko D, Dorsey SM, Nikou A, Burdick JA, Gorman JH, Jackson BM, Pilla JJ, Gorman RC, Wenk JF (2015). Temporal changes in infarct material properties: an in vivo assessment using magnetic resonance imaging and finite element simulations. Ann Thorac Surg.

[22] Xi J, Lamata P, Niederer S, Land S, Shi W, Zhuang X, Ourselin S, Duckett SG, Shetty AK, Rinaldi CA, Rueckert D, Razavi R, Smith NP (2013). The estimation of patient-specific cardiac diastolic functions from clinical measurements. Med Image Anal.

[23] Peskin CS (1975). Mathematical aspects of heart physiology.

[24] Axel L (2002). Biomechanical dynamics of the heart with MRI. Annu Rev Biomed Eng.

[25] Saber NR, Gosman AD, Wood NB, Kilner PJ, Charrier CL, Firman DN (2001). Computational flow modeling of the left ventricle based on in vivo MRI data: initial experience. Ann Biomed Eng.

[26] Guccione JM, McCulloch AD, Waldman LK (1991). Passive material properties of intact ventricular myocardium determined from a cylindrical model. J Biomech Eng.

[27] Krishnamurthy A, Villongco CT, Chuang J, Frank LR, Nigam V, Belezzuoli E, Stark P, Krummen DE, Narayan S, Omens JH, McCulloch AD, Kerckhoffs RC (2013). Patient-specific models of cardiac biomechanics. J Comput Phys.

[28] Guccione JM, McCulloch AD (1993). Mechanics of active contraction in cardiac muscle: Part I-Constitutive relations for fiber stress that describe deactivation. J Biomech Eng.

[29] Guccione JM, Waldman LK, McCulloch AD (1993). Mechanics of active contraction in cardiac muscle: Part II-Cylindrical models of the systolic left ventricle. J Biomech Eng.

[30] Kerckhoffs RCP, Healy SN, Usyk TP, McCulloch AD (2006). Computational methods for modeling cardiac electromechanics. Proc IEEE.

[31] McCulloch AD. Continuity 6 (a package distributed free by the National Biomedical Computation Resource). 2007. http://www.continuity.ucsd.edu.

[32] McCulloch AD, Waldman L, Rogers J, Guccione JM (1992). Large-scale finite element analysis of the beating heart. Crit Rev Biomed Eng.

[33] Pfeiffer E, Tangney JR, Omens JH, McCulloch AD (2014). Biomechanics of cardiac electromechanical coupling and mechanoelectric feedback. J Biomech Eng.

[34] Tang D, Yang C, Geva T, Del Nido PJ (2008). Patient-specific MRI-based 3D FSI RV/LV/Patch models for pulmonary valve replacement surgery and patch optimization. J Biomech Eng.

[35] Tang D, Yang C, Geva T, Del Nido PJ (2010). Image-based patient-specific ventricle models with fluid-structure interaction for cardiac function assessment and surgical design optimization. Prog Pediatr Cardiol.

[36] Tang D, Yang C, Geva T, Gaudette G, Del Nido PJ (2010). Effect of patch mechanical properties on right ventricle function using MRI-based two-layer anisotropic models of human right and left ventricles. CMES Comput Model Eng Sci.

[37] Tang D, Yang C, Geva T, Gaudette G, Del Nido PJ (2011). Multi-physics MRI-based two-layer fluid-structure interaction anisotropic models of human right and left ventricles with different patch materials: cardiac function assessment and mechanical stress analysis. Comput Struct.

[38] Fan L, Yao J, Yang C, Tang D, Xu D (2015). Infarcted left ventricles have stiffer material properties and lower stiffness variation: 3D echo-based modeling to quantify in vivo ventricle material properties. J Biomech Eng.

[39] Humphrey JD (2002). Cardiovascular solid mechanics.

[40] R Core Team. R: A Language and Environment for Statistical Computing, R Foundation for Statistical Computing. Vienna. 2014. http://www.R-project.org.

